# Attitudes Toward Psychological Disorders and Alternative Medicine in Saudi Participants

**DOI:** 10.3389/fpsyt.2021.577103

**Published:** 2021-02-12

**Authors:** Mohamed H. Alegiry, Nahid H. Hajrah, Nada A. Y Alzahrani, Hossam H. Shawki, Muhammadh Khan, Houda Zrelli, Ahmed Atef, Youngil Kim, Ibrahim A. Alsafari, Leila Arfaoui, Hesham F. Alharby, Abdulrahman S. Hajar, Hesham El-Seedi, Lekh Raj Juneja, Jamal S. M. Sabir, Abdelfatteh El Omri

**Affiliations:** ^1^Department of Biological Sciences, Faculty of Science, King Abdulaziz University (KAU), Jeddah, Saudi Arabia; ^2^Center of Excellence in Bionanoscience Research, King Abdulaziz University, Jeddah, Saudi Arabia; ^3^Department of Comparative and Experimental Medicine, Graduate School of Medical Sciences, Nagoya City University, Nagoya, Japan; ^4^National Gene Bank of Egypt (NGB), Agricultural Research Center (ARC), Giza, Egypt; ^5^Food Business Promotion Division, ROHTO Pharmaceutical Co., Ltd., Osaka, Japan; ^6^Department of Biology and Department of Chemistry, College of Science, University of Hafr Al Batin, Hafr Al Batin, Saudi Arabia; ^7^Clinical Nutrition Department, Faculty of Applied Medical Sciences, King Abdulaziz University, Jeddah, Saudi Arabia; ^8^Pharmacognosy Group, Department of Medicinal Chemistry, Biomedical Centre, Uppsala University, Uppsala, Sweden

**Keywords:** alternative medicine, psychological disorder, psychopharmacology, survey, Saudi participants

## Abstract

**Background:** This study was designed to investigate Saudis' attitudes toward mental distress and psychotropic medication, attribution of causes, expected side effects, and to analyze participants' expectations toward alternative or complementary medicine using aromatic and medicinal plants, through a survey.

**Method:** The study included 674 participants (citizens and residents in Saudi Arabia) who were randomly contacted via email and social media and gave their consent to complete a questionnaire dealing with 39 items that can be clustered in six parts. Descriptive statistics and Chi-square for cross-tabulation were generated using SPSS.

**Results:** Among the 664 participants, 73.4% believed that there are some positive and negative outcomes of psychotropic medication. Participants (72.0%) think that the most important reason leading to psychological disorders is mainly due to the loss of a relative or beloved person, and 73.9% considered psychic session as one of the possible treatments of psychological disorders. Surprisingly, only 18.8% of the participants agreed that medicinal and aromatic plants could be a possible treatment of the psychological disorder. Participants (82%) consider that physicians are the most trustful and preferred source of information about alternative and complementary medicine.

## Introduction

Mental health problems and disorders are becoming increasingly widespread throughout the world. The lifetime risk for any psychiatric disorder is ~50% (1). There appears to be a complex relationship between stressful situations, our mind, and the body's reaction to stress, and the onset of mental issues. The term “mental health literacy” was coined by Jorm et al. (2) It is defined as the “knowledge and beliefs about mental disorders, which help to promote, manage, and maintain good mental health” (1). It includes the ability to recognize specific disorders; knowing how to seek mental health information; knowledge of risk factors and sources of self-treatment and of professional help available; and attitudes that promote recognition and appropriate help-seeking (3). A multitude of factors such as socioeconomic status, isolation, discrimination (4), socio-demographic factors (5), race, and level of education (6), financial concerns, health problems, and communication difficulties (7) may affect mental health. How people come to understand their mental distress and attitude toward seeking professional help has been shown to be strongly rooted in wider cultural health beliefs (8, 9).

Recently, several strategies including education and communication have been designed to improve mental health literacy (3). However, few studies have been conducted to promote complementary and alternative medicine (CAM) based on aromatic and medicinal plants as an alternative for mental health care (10). CAM can be defined as “a group of diverse medical and health care systems, practices, and products that are not currently considered to be part of conventional medicine” (11).

The rising image of green consumerism, reduced faith in conventional treatments, and the growth in the availability of alternative remedies have contributed to the increased popularity of CAM around the world. In the United States of America, it was revealed through a national survey that the majority of CAM users are dissatisfied with conventional medicine and they consider CAM to be more congruent with their own values, beliefs, and philosophical orientation toward health and life (12). In Saudi Arabia, several traditional remedies are used for mental health care; although such practices are not well-documented and need to be sustained. To the best of our knowledge, there has been no study that examines Saudis' attitude toward mental distress and the professional use of aromatic and medicinal plants as CAM. Therefore, we conducted a survey among Saudi people to investigate the public's attitude toward mental distress problems and psychotropic drug treatment.

Locally, the percentage of Saudis who practice CAM lies between 33 and 93% (13). The knowledge of CAM derives mainly from family, friends, and media, while religion and local culture play a crucial role in enhancing the knowledge and the number of practitioners within the community. A limited number of studies have been published with regard to the use of CAM by patients in Saudi Arabian hospitals. However, a study has shown that there are various reasons that have driven cancer patients to use CAM as a treatment, whether it be as a mood enhancer, for pain control, as an immune system enhancer, for fitness purposes, or as an appetite enhancer (13). Not only has CAM been reported to be used among cancer patients, but also pregnant women have turned to it during pregnancy, labor, and post-birthing. In a study by Sameer et al., it was reported that 25.3% of Saudi women who participated in their study acknowledged the use of CAM during pregnancy, while a higher percentage used CAM during labor and almost half of the participants used CAM after delivery (14).

## Methods

### Study Design

The study was approved by the department of Biological Sciences at King Abdulaziz University as a part of a funded academic research. Participants were randomly contacted via social media and they were asked to give their consent to voluntarily participate to the current academic study without giving any detail about their personal details like names, address, email, phone number, among others. Those who did not agree were withdrawn from the study. Collected data was checked and coded by the principal investigators prior to data analysis.

#### Socio-Demographic Characteristics

Participants were asked to provide information on their age, gender, level of education, job, marital status, and living status. With regard to their cultural background, participants were asked to select their region of origin and to select whether they were Saudi's citizens or residents. In addition, they were asked to select their monthly income in Saudi Riyal (SR).

#### Beliefs About Psychological Disorders

The participants' attitude toward mental distress and the relationship between their background and actual state of chronic disease was surveyed in the beliefs about psychological disorders (BPD) questionnaire. Questions about the recognition of the existence of a psychological disease and the individual's experience with mental distress were included. The first question was “Have you ever experienced signs and symptoms related to psychological disorders such as depression, anxiety, or stress?” Additional questions were about the frequency of these signs and symptoms. The first question was a Yes/No question. The next question dealt with the frequency of the experience psychological issue using the following scale: very often, occasionally, rarely, and never.

The next section mainly concerned the individual's experience regarding medications for physical or mental chronic disease and their side effects on the body and behavior. Answers ranged from positive effects, negative effects, no effects at all, or had neither positive nor negative side effects.

#### Attribution of Causes of Psychological Disorder and Potential Remedies

The attribution of causes of psychological disorder (ACPD) questionnaire was designed to assess the individual's attributions of cause and some possible remedies for psychological disorders. The APCD questionnaire included nine statements, which examined attributions reflecting (i) biological factors, (ii) psychosocial factors (work, study, or stressful events), (iii) environment factors, and (iv) supernatural factors (The evil eye, magic, astrology), which lead to mental distress, and the participants were also asked to add other possible causes that might lead to mental distress. These causal attribution dimensions were based on constructs in the studies of Al-Krenawi et al. (6). Possible remedies for psychological disorders in the survey included six statements, which investigated the preferred treatment approach such as relying on personal resources for help, pharmacotherapy medication, psychological consulting, meditation, diet, medicinal and aromatic plants, religious activities, and practicing sports. In addition, the participant was given the opportunity to add other remedies for the psychological disorders. The items were rated on a 3-point scale (yes = 1, maybe = 2, no = 3).

#### Expected Effects of Psychotherapy Medications and Perceived Influence of Ethnocultural Context

This questionnaire included six statements and was adapted from a previous study by Thorens et al. (9). It investigated the expected side effects of psychotropic agents by patients (positive or negative changes in character or attitude), interference of treatment with religious beliefs, and opinions of the individuals surrounding the patient, and the use of alternative medicines (complementary medicine, homeopathy, and traditional medicines). These items were given as Yes/No questions.

#### People's Opinion About Medicinal and Herbal–Based Functional Food Formulations

This questionnaire consists of four statements. Patients were requested to answer using yes or no responses regarding their preferred source of information about these products (doctor, herbalist, nutritionist, family, fellow patient, books and magazines, television, and the internet). We also asked participants to add another source of information. Finally, we added an open question related to the individual's suggestions about plants or herbs used traditionally for psychotherapy.

### Study Participants

Participants were recruited through electronic media using several platforms. The study was conducted using a questionnaire written in Arabic from October 1st to October 17th, 2018. The participants included 674 adults from different Saudi regions.

### Data Collection, Measures, and Statistical Analysis

#### Survey

Two preliminary versions were tested, thus permitting to reformulate or eliminate questions that were difficult to understand or ambiguous. The questionnaire dealt with the individual's concept of mental distress, attribution of causes, and remedies for psychological disorders. Several studies were conducted to predict treatment-seeking behavior across different ethnic groups, and across various age groups, including students and other adults, by administering self-reported questionnaires.

#### Data Analysis

Chi-square was calculated and significance were considered at *p* < 0.05. Data were analyzed using the IBM SPSS Version 25.0, 2017.

## Results

### Socio-Demographics of Participants

As shown in Table 1, a total of 674 participants agreed to participate in the survey with good male/female diversity: 304 (45.1%) males and 370 (54.9%) females. The majority of the participants were from the central region, which included the capital of Saudi Arabia (57.4%) followed by the western region (20.6%). Few participants responded to the survey from the other regions: 8.9% from the Eastern region, 6.4% from the Northern Region, 4.3% from the Southern Region, while very few participants (2.4%) originated from regions outside the Kingdom of Saudi Arabia. The majority of the participants were aged 18–30 years old (41.1%), while the fewest were over age 60 years (4.6%). Participants aged 31–40, 41–50, and 51–60 years old were 22.8, 16.0, and 15.4%, respectively. Most participants were Saudis (98.2%), with only (1.8%) from other nationalities. Over half of the participants were married (56.4%), while 39.9% of participants were single. The martial status with the fewest participants were divorced (2.7%) and widowed (1.0%). The participants who lived as a couple were 56.2%, while 37.7% responded that they still lived with their parents. Few of the participants lived alone or with roommates (4.5 and 1.6%, respectively). The majority of participants held a bachelor's degree (69.6%), while 11.6% of the total participants held higher degrees. The remaining 16% of the participants held a secondary school certificate, and the fewest participants indicated a middle school certificate as their educational level (2.8%). The occupations of the participants differed greatly, and included employees (24.7%), housekeepers (15.3%), retirees (14.4%), or students (13.8%). Also included were unemployed participants (8.9%) and merchants (1.3%). Financially, participants whose monthly income ranged between 11,000 and 20,000 SR were 29.1%, while participants having a monthly income ranging between 5,000 and 10,000 SR, and <5,000 SR were 25.4 and 25.5%, respectively. Finally, 18.8% of participants had a monthly income >20,000 SR.

**Table 1:** The distribution of participants by gender, age, region, marital and living status, education, income, and occupation.

**Variable**		***n* (%)**
Gender	Men	304 (45.1%)
	Women	370 (54.9%)
Region of origin	Central Region	387 (57.4%)
	Eastern Region	60 (8.9%)
	Northern Region	43 (6.4%)
	Southern Region	29 (4.3%)
	Western Region	139 (20.6%)
	Outside the kingdom	16 (2.4%)
Age	18–30	277 (41.1%)
	31–40	154 (22.8%)
	41–50	108 (16.0%)
	51–60	104 (15.4%)
	Over 60	31 (4.6%)
Nationality	Saudi	662 (98.2%)
	Resident	12 (1.8%)
Marital status	Single	269 (39.9%)
	Married	380 (56.4%)
	Divorced	18 (2.7%)
	Widowed	7 (1.0%)
Living status	Alone	30 (4.5%)
	Couple	379 (56.2%)
	Family	254 (37.7%)
	Roommate	11 (1.6%)
Education	Middle school	19 (2.8%)
	Secondary	108 (16%)
	University	469 (69.6%)
	Higher education	78 (11.6%)
Job	Government Employee	167 (24.7%)
	Housekeeper	103 (15.3%)
	Merchant	9 (1.3%)
	Retired	97 (14.4%)
	Student	93 (13.8%)
	Unemployed	60 (8.9%)
Monthly income	11,000–20,000	196 (29.1%)
	5,000–10,000	171 (25.4%)
	<5,000	172 (25.5%)
	More than 20,000	127 (18.8%)

### Perception About Experiencing Psychological Disorder Symptoms (BPD)

As shown in Table 2, participants were asked if they had experienced signs or symptoms related to psychological disorders. The majority of the participants acknowledged that they had experienced these signs (71.2%), while 28.8% of the participants said they have not experienced any signs or symptoms related to psychological disorders. For 31.1% of the participants, the intensity and the frequency of these signs were reported as “sometimes,” while 28% described symptom occurrence as “rarely” and 12.5% as “often.”

**Table 2:** Participants'previous psychological disorder events and their frequency.

	**Answer (*****n*****, %)**						
**Question**	**Yes**	**No**	**Never**	**Often**	**Rarely**	**Sometimes**	**Total**
Previous experience of psychological disorders signs	480 (71.2%)	194 (28.8%)	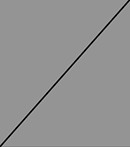	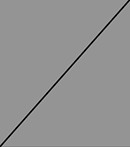	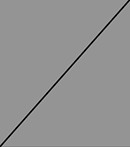	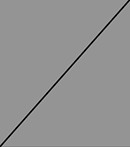	674 (100%)
Frequency of experienced psychological disorders	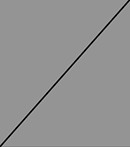	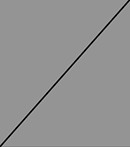	190 (28.2%)	84 (12.5%)	189 (28.0%)	211 (31.3%)	674 (100%)

### Expected Effects of Psychotherapy Medication, and Perceived Influence of Ethnocultural Context

As shown in Table 3, among the 664 participants, the majority (73.4%) believed that there were both positive and negative effects of psychotropic drugs on the treatment of psychological disorders, while 16.3% of the participants believed that there were only negative effects due to these drugs. Only 5.0% of participants believed that psychotropic drugs exerted positive effects, while 3.7% of the participants believed that these drugs did not have any therapeutic effects at all. With regard to the effects of psychotropic drugs on behavior and personality of the patients, 68.7% of the participants believed that there were both negative and positive effects on behavior and personality, while 14.2% of the participants believed that these drugs only exerted negative effects. Overall, 12.0% of the participants believed that psychotropic drugs had a positive effect on behavior and personality, and only 2.7% felt that there was no effect of these psychotropic drugs on the behavior and personality of the patients. Participants were also asked about the impact of the psychotropic drugs on the general functions of the human body: 47.9% stated that these agents had either a positive and negative influence on the general functions of the human body, while 36.6% believed that they had only negative effects. Participants believing that psychotropic drugs exerted positive effects on the general functions of the human body represented 4.3% of the total participants, while 9.5% of the participants felt that they had no effect.

**Table 3:** Participants attitude toward psychotropic medication outcomes in general and their effect on behavior and body function in human.

**Question**	**Participants answers (*****n*****, %)**				
	**Negative effect**	**No effect**	**Positive and Negative effect**	**Positive effect**	**Total**
Attitude of participants toward psychotropic medication outcomes in patients	110 (16.3%)	25 (3.7%)	**495** **(73.4%)**	34 (5.0%)	664 (98.5%)
Attitude of participants toward psychotropic medication effects on behavior and personality	96 (14.2%)	18 (2.7%)	**463** **(68.7%)**	85 (12.0%)	662 (98.2%)
Attitude of participants toward psychotropic medication effects on human organ functions	247 (36.6%)	64 (9.5%)	**323 (47.9%)**	29 (4.3%)	663 (98.4%)

### Attribution of Causes of Psychological Disorder and Possible Remedies

As shown in Table 4, the most important causes of psychological disorders according to the participant's perspective were losing someone close (72.0%), stress form work and school (71.8%), and social responsibilities (70.2%). Almost half of the participants believed that a biological disorder was one of the most important causes of psychological disorders (50.6%). Environmental factors and genetics constituted the most important causes of psychological disorders according to 45.4% and 45.3% of the participants, respectively. In addition, 36.6% of the participants agreed that Black magic was a potential cause, while (36.5%) of participants agreed that the evil eye could be an attributed cause. Only 6.7% of the participants said that astrology leads to psychological disorders.

**Table 4:** Participants perception and beliefs about the most important factor leading to psychological disorders.

**Possible factor**	**Yes**	**Maybe**	**No**	**Total**
Biological factors	341 (50.6%)	307 (45.5%)	24 (3.6%)	672 (99.7%)
Environmental factors	306 (45.4%)	270 (40.1%)	96 (14.2%)	672 (99.7%)
Genetics	305 (45.3%)	264 (39.2%)	100 (14.8%)	669 (99.3%)
Work or education stress	484 (71.8%)	162 (24.0%)	25 (3.7%)	671 (99.6%)
Social responsibility	473 (70.2%)	173 (25.7%)	25 (3.7%)	671 (99.6%)
Losing someone close	485 (72.0%)	161 (23.9%)	24 (3.6%)	670 (99.4%)
Evil eye	246 (36.5%)	269 (39.9%)	155 (23.0%)	670 (99.4%)
Black magic	247 (36.6%)	249 (36.9%)	174 (25.8%)	670 (99.4%)
Astrology	45 (6.7%)	127 (33.1)	496 (73.6%)	668 (99.1%)

### Participant's Opinions on Medicinal and Herbal–Based Functional Food Formulations

As shown in Table 5, 73.9% of the participants considered a psychic session could represent a possible treatment for psychological disorders, while 72.3% said that physical activities and sport are a possible treatment. In addition, 63.9% believed that religious activities were a possible treatment for psychological disorder, while prescribed medications (60.4%), diet (33.8%), spiritual exercise (23.1%), and medicinal and herbal plants (18.8%) also represented treatment alternatives.

**Table 5:** Participants perception and beliefs about the possible treatment of psychological disorders.

**Treatment**	**Yes**	**Maybe**	**No**	**Total**
Prescribed medication	407 (60.4%)	223 (33.1%)	38 (5.6%)	668 (99.1%)
Psychic session	498 (73.9%)	144 (21.4%)	25 (3.7%)	667 (99.0%)
Medicinal and aromatic plants	125 (18.8%)	346 (51.3%)	193 (28.6%)	664 (98.5%)
Religious activities	431 (63.9%)	178 (26.4%)	58 (8.6%)	667 (99.0%)
Spiritual exercise	156 (23.1%)	324 (48.1%)	189 (28.0%)	669 (99.3%)
Diet	228 (33.8%)	314 (46.6%)	124 (18.4%)	666 (98.8%)
Workout	487 (72.3%)	164 (24.3%)	19 (2.8%)	670 (99.4%)

As shown in Table 6, the majority of participants chose physicians as the preferred source of information for the usage of medicinal plants to treat psychological disorders (81.8%), while (63.9%) of the participants considered nutritionists as the preferred source of information. In addition, other patients (47.3%), the internet (46.9%), family members (36.4%), and herbalists (34.3%) were the preferred sources of information for treating psychological disorders. Books and magazines were the preferred sources for 30.0% of the participants, while TV was the least preferred source of information for 25.2% of participants.

**Table 6:** Participants perception and beliefs about their preferred source of information to treat psychological disorders by medicinal plants.

**Source**	**Yes**	**No**	**Total**
Physician	551 (81.8%)	108 (16.0%)	659 (97.8%)
Nutritionist	431 (63.9%)	229 (34.0%)	660 (97.9%)
Herbalist	231 (34.3%)	429 (63.6%)	660 (97.9%)
Family	245 (36.4%)	412 (61.1%)	657 (97.5%)
Patients	319 (47.3%)	341 (50.6%)	660 (97.9%)
Books and magazine	202 (30.0%)	458 (68.0%)	660 (97.9%)
TV	170 (25.2%)	486 (72.1%)	656 (97.3%)
Web	316 (46.9%)	337 (50.0%)	653 (96.9%)

As shown in Table 7, our results showed that the gender of the participants was significantly correlated with experiencing symptoms related to psychological disorders, although, there was no significant correlation in terms of the intensity of these symptoms. The age of the participant was significantly correlated with experiencing symptoms and their intensity and on the participant's perspective on the side effects of the drugs and on its effects on the normal function of the human body (Table 7 and Supplementary Material 1).

**Table 7:** Cross tabulation between participants' socio-demographic parameters and different survey question.

**Question**		**Pearson Chi-Square significance**								
		**Gender**	**Age**	**Nationality**	**Residence region**	**Education**	**Occupation**	**Social Status**	**Living Status**	**Income**
Previous or current experience of psychological disorder	Yes/No	0.004	0.000	0.770	0.004	0.215	0.011	0.000	0.000	0.063
	Frequence	0.062	0.000	0.759	0.182	0.019	0.000	0.000	0.000	0.046
Attitude toward psychotropic medication outcomes and effect on	In patients	0.432	0.006	0.500	0.465	0.680	0.009	0.036	0.031	0.498
	On behavior and personality	0.429	0.101	0.493	0.230	0.266	0.576	0.019	0.003	0.130
	On human organ functions	0.012	0.009	0.068	0.942	0.003	0.054	0.039	0.001	0.003
Participants perception and beliefs about the most important factor leading to psychological disorders	Biological factors	0.030	0.001	0.483	0.263	0.151	0.402	0.012	0.171	0.956
	Environmental factors	0.593	0.000	0.333	0.116	0.003	0.001	0.000	0.000	0.438
	Genetics	0.203	0.000	0.334	0.025	0.018	0.000	0.004	0.000	0.011
	Work or Education stress	0.198	0.000	0.337	0.013	0.003	0.051	0.000	0.000	0.145
	Social responsibility	0.451	0.022	0.567	0.409	0.084	0.007	0.007	0.012	0.039
	Losing someone close	0.006	0.000	0.011	0.002	0.010	0.005	0.004	0.002	0.577
	Evil eye	0.663	0.000	0.969	0.000	0.142	0.004	0.000	0.001	0.737
	Black magic	0.692	0.046	0.603	0.005	0.041	0.218	0.033	0.186	0.880
	Astrology	0.435	0.062	0.358	0.279	0.923	0.393	0.533	0.867	0.633
Participants perception and beliefs about the treatment of psychological disorders	Prescribed medication	0.4107	0.652	0.921	0.375	0.875	0.517	0.974	0.617	0.410
	Psychic session	0.1282	0.005	0.663	0.252	0.000	0.037	0.012	0.047	0.000
	Medicinal and aromatic plants	0.0189	0.002	0.621	0.003	0.676	0.007	0.315	0.525	0.234
	Religious activities	0.3823	0.019	0.473	0.001	0.320	0.100	0.001	0.008	0.844
	Spiritual exercise	0.0079	0.010	0.840	0.104	0.040	0.182	0.000	0.001	0.532
	Diet	0.169	0.043	0.383	0.853	0.548	0.186	0.012	0.145	0.141
	Workout	0.5061	0.773	0.361	0.460	0.396	0.971	0.310	0.687	0.462
Participants perception and beliefs about their prefer resource of information to treat psychological disorders by medicinal plants	Physician	0.4789	0.364	0.416	0.105	0.339	0.541	0.233	0.123	0.642
	Nutritionist	0.0022	0.000	0.261	0.070	0.081	0.014	0.019	0.021	0.414
	Herbalist	0.082	0.002	0.464	0.007	0.833	0.011	0.000	0.000	0.175
	Family	0.5987	0.437	0.358	0.875	0.138	0.006	0.020	0.013	0.182
	Patients	0.2021	0.447	0.641	0.491	0.880	0.148	0.676	0.270	0.044
	Books and magazine	0.2354	0.140	0.671	0.010	0.807	0.051	0.217	0.298	0.119
	TV	0.3525	0.024	0.039	0.158	0.885	0.054	0.001	0.009	0.037
	Web	0.0039	0.531	0.005	0.441	0.982	0.257	0.857	0.684	0.045

*Pearson Chi-square is significant at p <0.05. Details of cross tabulation are available in the Supplementary Material 1*.

The region of residence of the participants was correlated with experiencing symptoms of psychological disorders. Furthermore, the educational level of the participants significantly correlated with the intensity of the psychological episode and with the participant's perspective of the impact of the psychotropic drugs on the normal function of the human body (Table 7 and Supplementary Material 1).

Occupation also showed a significant correlation with experiencing symptoms and their intensity, and also with participant's perspective on the side effects of the psychotropic agent. Furthermore, social status and living conditions of the participants were also significantly correlated with experiencing symptoms and their intensity, as were their perspective on the side effects of the medications and their influence on behavior and the normal function of the human body. The income of the participants also showed a significant correlation with the intensity of the symptoms and the participant perspectives on the impact of the psychotropic medications reflections on the normal function of the human body (Table 7 and Supplementary Material 1).

The participants' gender was also significantly correlated with biological factors and losing someone close as the most important causes leading to psychological disorders, while the age of the participant was significantly correlated with considering biological factors, environmental factors, genetics, stress from work or school, social responsibility, losing someone close, the evil eye, and black magic as the most important causes leading to psychological disorders. The nationality of the participants was significantly correlated only with considering losing someone close as the most important reason leading to a psychological disorder (Table 7 and Supplementary Material 1).

The educational level of the participants significantly correlated with choosing environmental factors, genetics, stress from work or school, losing someone close, and black magic as the most important causes leading to psychological disorders, while the participant's occupation significantly correlated with environmental factors, genetics, social responsibility, losing someone close, and the evil eye as causes of psychological disorders (Table 7 and Supplementary Material 1).

The perspective of surveyed participants with regard to potential treatments for psychological disorders was significantly correlated with the gender of the participant and also with considering medicinal and herbal plants and spiritual exercises as possible treatments, while the age of the participants correlated with choosing psychic sessions, religious activities, spiritual exercises, medicinal and herbal plants, and diet as possible forms of treatment. The educational level of the participants was significantly correlated with believing that psychic sessions and spiritual activities were possible treatments for psychological disorders, while occupation was correlated with considering psychic sessions and medicinal and herbal plants as possible treatments. The social status of the participants was significantly correlated with considering a psychic session, religious activities, spiritual exercise, and diet as possible treatments, while the living conditions of the participants was correlated with choosing psychic sessions, religious activities, and spiritual exercises as potential treatments. Monthly income correlated only with considering psychic sessions as a possible treatment (Table 7 and Supplementary Material 1).

The participants' perspective on their preferred source of information regarding medicinal plants to treat psychological disorders was significantly correlated with gender, age, region of residence, occupation, social status, living conditions, and monthly income of the participants. The gender of the participant was also correlated with considering nutritionists and the internet as preferred sources of information, while the age of the participants correlated with choosing nutritionists, herbalists, and the TV as sources of information. The participants' region of origin correlated with considering herbalists, and books and magazines, while the occupation of the participants showed a significant correlation with nutritionists, herbalists, and family as sources of information about medicinal plants to treat psychological disorders. Social and living status of the participants were correlated with considering nutritionists, herbalists, and family as sources of information (Table 7) and for more details about the cross tabulation between the sociodemographic parameters and the different survey question refer to the Supplementary Material 1.

The participants were asked to suggest plants or herbs that they knew were traditionally used in psychotherapy. As shown in Table 8, the participants reported a total of 39 plants (Table 8, and references therein).

**Table 8:** Participants suggestions about plants and herbs used traditionally for psychotherapy.

**Plant**	**Latin name**	**Frequency (Number of response)**	**Neuro-protective**	**Anxiolytic**	**Anti-depressant**	**Anti-stress**
Mint	*Mentha sp*.	32	(22)	(23)	(24)	(25)
Chamomile	*Chamaemelum nobile*	22		(27)		
Anise	*Pimpinella anisum*	16	(31)	(29)	(30)	
Lavender	*lavandula sp*.	11	(33)	(33)	(33)	
Safflower	*Carthamus tinctorius*	11	(35)	(36)	(36)	
Barley	*Hordeum vulgare*	8	(37)		(38)	(39)
Lemon	*Citrus limon*	7	(40)	(41)	(42)	
Rose blossom water	*Rosa sp*.	6				
Marijuana	*Cannabis sativa*	5	(43)	(44)	(45)	(46)
Coffee	*Coffea arabica*	4	(47)			
Ginger	*Zingiber officinale*	4	(48)	(49)	(50)	
Sage	*Salvia officinalis*	3	(51)	(52)	(52)	
Saffron	*Crocus sativus*	3	(53)	(54)	(55)	
Rosemary	*Rosmarinus officinalis*	3	(56)	(57)	(57)	
Saint John's wort	*Hypericum perforatum*	3	(58)	(59)	(60)	(61)
Myrrh	*Commiphora myrrha*	3	(62)			
Nigella	*Nigella sativa*	3	(63)	(64)	(64)	
Jasmin	*Jasminum sp*.	2			(65)	
Chocolate	*Theobroma cacao*	2	(66)	(67)	(68)	
Valerian	*Valeriana officinalis*	2	(69)	(70)	(71)	
Costus or Kuth	*Saussurea costus*	2		(72)		
Basil	*Ocimum basilicum*	1	(73)	(74)	(75)	
Banana	*Musa sp*.	1			(76)	
Ziziphus	*Ziziphus sp*.	1	(77)	(78)		
Tea	*Camellia sinensis*	1	(79)	(80)	(80)	
Caraway	*Carum carvi*	1				
Shamer-Fennel	*Foeniculum vulgare*	1		(81)	(81)	(82)
Senna	*Senna alexandrina*	1				
Kava kava	*Piper methysticum*	1	(83)	(84)	(85)	
Cumin	*Cuminum cyminum*	1	(86)	(87)		(88)
Olive oil	*Olea europaea*	1	(89)	(90)	(90)	
Oregano	*Origanum majorana*	1	(91)	(92)	(93)	
Melberry	*Morus nigra*	1	(94)		(94)	
Kady water	*Pandanus tectorius*	1		(95)	(95)	
Pumpkin	*Cucurbita pepo*	1	(96)		(97)	
Carotts	*Daucus carota*	1			(98)	
Hibiscus	*Hibiscus rosa-sinensis*	1	(99)	(100)	(101)	
Beet roots	*Beta vulgaris*	1		(102)	(102)	
Hilteet	*Asafoetida*	1	(103)	(103)	(104)	

## Discussion

The younger population is exposed to significant stress from social interactions, school, and work. Moreover, rapid changes are occurring in the global society due to technological expansion, which influences the quality of mental health (15). Thus, it is expected that a significant proportion of the respondents (71.2%) in our study are individuals who have experienced signs of psychological disorders such as depression or anxiety.

Male and females were equally distributed among the responders to our survey. However, the results showed a significantly higher correlation between the gender of the participant and experiencing of symptoms relating to psychological disorders. In this study, female participants (58.5%) more frequently experienced these symptoms than male participants (41.5%). Hormonal fluctuation is a possible factor that contributes to the increased prevalence of psychological disorders like depression among women, the global prevalence of depression in 2010 was 5.5% in women, while the prevalence among men was 3.2% (16).

The level of household income has been associated with the presence of mental disorders in some studies, while other studies have shown no association. A reduction of household income is associated with an increased risk for incident mental disorders (17). In our study, the monthly income of the participants was correlated with the intensity of the signs of psychological disorders: 38.1% of the participants who described the intensity of their signs of psychological disorder as “often” were from the lowest monthly income group while this percentage had an inverse relationship with the increase in monthly income.

In a study on postpartum depression in the western region of Saudi Arabia, among the causes and risk factors of postpartum depression, the least picked reasons by the participants were paranormal and supernatural causes such as the evil eye, ghosts, magic, and committing sins (18). In our study, the three least picked reasons chosen were black magic, the evil eye, and astrology. Some of these causes are based on a religious background, while others have a cultural background. The majority of the participants did not choose any of these reasons as the most important cause leading to psychological disorders. This reflects the perspective of the Saudis toward paranormal and supernatural events vs. reasons backed by logic and scientific reasoning in relation to human psychology.

Medicinal and herbal plants were the least picked agents for treatment of psychological disorders by the Saudis which reflects the low confidence in medicinal plants as a possible treatment option. In contrast, Saudis seem to have some belief in the application of alternative medicine and medicinal plants during pregnancy, post-delivery, and for some types of cancer symptoms (13, 14). In psychology-related disorders, our data showed doubts about the efficacy and safety of using alternative medicine and medicinal plants.

The results indicated a correlation between considering possible treatments for psychological disorders and gender, age, region of origin, educational level, occupation, social status, living conditions, and monthly income of the participants. However, the gender of the participant correlated significantly with considering spiritual activity as a possible treatment for psychological disorders, where 27.8% of the female participants believed in spiritual exercises compared to 17.7% of the male participants. While meditation could represent one of the spiritual exercises, the study shows that meditation might be more beneficial to females than to males due to differences in the mechanism of emotion regulation (19). This factor might explain why female participants showed a stronger belief in spiritual exercises than males in our study.

The 39 plants and herbs that were reported by the participants were a good start to building a local library of medicinal plants traditionally used for psychotherapy. Mint (*Mentha* sp.) was the most frequently cited plant among the answers, mentioned 32 times. Followed by chamomile (*Chamaemelum nobile*) suggested 22 times, anise (*Pimpinella anisum*) 16, lavender (*lavendula sp*.), and safflower (*Carthamus tinctorius*), 11 each. Most of the reported plants and herbs have been documented for their psychological properties such as neuroprotective, anxiolytic, anti-depressant, and anti-stress properties by its phytochemicals and secondary metabolites.

Mint (*Mentha sp*.) and its essential oils are popularly used in food, cosmetics, and pharmaceutical industries (20). Despite the different species of the plant, several studies have named carvone as its main active component (21). *Mentha sp*. has multiple biological activities that enhance its presence in CAM, and is used as a carminative, antispasmodic, diuretic, antibacterial, antifungal, and antioxidant agent. It has also been used to treat colds and flu, respiratory tract problems, gastralgia, hemorrhoids, and stomachache (20). As psychological treatment, several studies have reported that Menthe (*Mentha* sp.) has a neuroprotective, anxiolytic, anti-depressant, and anti-stress properties (22–25).

Chamomile (*Chamaemelum nobile*) dried flowers and its essential oil have been used in food and cosmetics. It has also been used as treatment for fever, inflammation, muscle spasms, gastrointestinal disorders, rheumatic pain, hemorrhoids, and other pathologies (26). Among the psychological properties, it has been reported that Chamomile (*Chamaemelum nobile*) has anxiolytic properties due to the presence of apigenin, which binds to central benzodiazepine receptors (27).

Anise (*Pimpinella anisum*) and its essential oil are used as flavoring and as an aromatic agent in food and has been used also as a digestive, carminative, and for gastrointestinal problems. Trans-anethole, estragole, γ-hymachalen, panisaldehyde, and methyl chavicol are considered the main constituents of the plant's oil (28). It has been reported that the extract of Anise seeds has an anxiolytic effect (29). In addition, the aqueous extract shows antidepressant-like effects (30). Its oil shows neuroprotective advantages as well (31).

Lavender (*lavandula sp*.) oil exerts antimicrobial activity *in vitro* and spasmolytic activity *in vivo* (32). It also exerts anxiolytic, anti-depressant-like effects, and neuroprotective properties (33).

Safflower (*Carthamus tinctorius*) has been used in food and traditional medicine due to its active compounds such as flavonoids, phenylethanoid glycosides, coumarins, fatty acids, and steroids to treat conditions such as dysmenorrhea, amenorrhea and other health issues (34). It has been shown that Safflower (*Carthamus tinctorius*) petal extract exerts neuroprotective, antidepressant, and antianxiety activities (35, 36). The list of suggested plants and their respective psychological properties are listed in Table 8.

## Conclusion

This study revealed the surveyed participants' perceptions and beliefs about psychological disorder in general and their experience about using medicinal plants to treat mental distress. Surveyed Saudis in the study have shown good knowledge about complementary and alternative medicine. However, they have some concerns about its efficacy and safety. Moreover, they consider that physicians are the most trustful to prescribe aromatic and medicinal plants as a part of psychotherapy.

## Data Availability Statement

The raw data supporting the conclusions of this article will be made available by the authors, without undue reservation.

## Ethics Statement

Ethical review and approval was not required for the study on human participants in accordance with the local legislation and institutional requirements. The patients/participants provided their written informed consent to participate in this study.

## Author Contributions

MA: conceptualization, data curation, formal analysis, methodology, writing—original draft, and review and editing. NH: conceptualization, data curation, funding acquisition, methodology, project administration, and review and editing. NA: data curation, formal analysis, methodology, and writing—original draft. HS: conceptualization, validation, methodology, and writing—original draft. MK and IA: formal analysis, methodology, validation, and review and editing. HZ: conceptualization, formal analysis, methodology, validation, and writing—original draft. AA and LA: formal analysis, methodology, validation, and writing—review and editing. YK: conceptualization, data curation, methodology, validation, and review and editing. HA and AH: formal analysis, funding acquisition, methodology, supervision, validation, and writing—review and editing. HE-S: conceptualization, validation, and review and editing. LJ: conceptualization, methodology, validation, and review and editing. JS: conceptualization, funding acquisition, methodology, project administration, supervision, validation, and review and editing. AE: conceptualization, data curation, formal analysis, funding acquisition, methodology, project administration, supervision, validation, writing—original draft, and review and editing. All authors contributed to the article and approved the submitted version.

## Conflict of Interest

YK and LJ were employed by the company Rohto Pharmaceuticals. The remaining authors declare that the research was conducted in the absence of any commercial or financial relationships that could be construed as a potential conflict of interest.
